# Lumbar Degenerative Disc Disease: Current and Future Concepts of Diagnosis and Management

**DOI:** 10.1155/2012/970752

**Published:** 2012-04-02

**Authors:** Fadi Taher, David Essig, Darren R. Lebl, Alexander P. Hughes, Andrew A. Sama, Frank P. Cammisa, Federico P. Girardi

**Affiliations:** Department of Orthopedic Surgery, Spine Service, Hospital for Special Surgery, New York, NY 10021, USA

## Abstract

Low back pain as a result of degenerative disc disease imparts a large socioeconomic impact on the health care system. Traditional concepts for treatment of lumbar disc degeneration have aimed at symptomatic relief by limiting motion in the lumbar spine, but novel treatment strategies involving stem cells, growth factors, and gene therapy have the theoretical potential to prevent, slow, or even reverse disc degeneration. Understanding the pathophysiological basis of disc degeneration is essential for the development of treatment strategies that target the underlying mechanisms of disc degeneration rather than the downstream symptom of pain. Such strategies ideally aim to induce disc regeneration or to replace the degenerated disc. However, at present, treatment options for degenerative disc disease remain suboptimal, and development and outcomes of novel treatment options currently have to be considered unpredictable.

## 1. Introduction

Low back pain (LBP) is the single most common cause for disability in individuals aged 45 years or younger and as a result carries tremendous weight in socioeconomic considerations. National economic losses resulting from LBP are estimated to exceed $100 billion per year and are mainly indirect due to reduced productivity [1]. Even though radiographic signs of degenerative disc disease (DDD) have been shown in asymptomatic individuals [2] and the degree of degeneration is by no means a marker for duration or severity of symptoms associated to DDD, ways of limiting disc degeneration or even inducing disc regeneration are still desirable goals in its treatment.

Strategies for stopping or reversing disc degeneration in the lumbar spine range from mechanical treatment options, that rely on the traditional concept of removing the pain generator, the disc, and eliminating pain by stopping motion, to more recently emerging and developing treatment options involving gene therapy, growth factors, and cell transplantations. The traditional approach of motion-eliminating fusion surgery, which may be effective for the treatment of pain in some cases, may also increase the rate of degeneration at adjacent spinal motion segments. Furthermore, this strategy does not halt the progression of the degenerative cascade of events that leads to pain and disability. So despite its undeniable significance, lumbar fusion surgery as a treatment of LBP has to be regarded suboptimal, as it targets the symptom of pain rather than its causes. The modern molecular biology era has brought revolutionary advances in fields such as genomics, nanotechnology, stem cell biology, gene therapy, and tissue engineering, which together hold tremendous therapeutic potential for clinical applications in degenerative disorders such as DDD.

## 2. Pathophysiology of Disc Degeneration

### 2.1. Anatomy and Innervation of the Intervertebral Disc

The intervertebral disc (IVD) is composed of the nucleus pulposus (NP) centrally, the annulus fibrosus (AF) peripherally, and the cartilaginous endplates cranially and caudally at the junction to the vertebral bodies. Within the NP, an abundance of proteoglycans allows for absorption of water. This property of the NP is essential for the IVD's handling of axial loads. In the healthy disc, the most common type of collagen within the NP is type II collagen. The AF surrounds the NP and consists primarily of type I collagen.

Descriptions of the innervation of the IVD have been published more than 20 years ago [3]. Branches of the sinuvertebral nerve, the spinal nerves, and gray rami communicantes [4] are believed to be part of the neurologic basis for discogenic back pain. An increase of nerve fibers and blood vessels in the painful disc, reaching regions of the annulus fibrosus and nucleus pulposus that are usually aneural in the healthy disc, has been reported, and a correlation between these findings and the expression levels of neurotrophins has been suggested [5].

### 2.2. Aging and Degeneration

The process of degeneration compares to the process of aging in many ways. However, disc degeneration often occurs at a faster rate, making DDD a condition often encountered in patients of working age. Quantitative gene expression analysis in a rabbit model suggests age to contribute uniquely to the degeneration process when compared to an injury-induced degeneration model [6]. With increasing age, the water content of the IVD decreases and fissures in the NP, potentially extending into the AF, can occur, and the start of this process, termed chondrosis intervertebralis, can mark the beginning of degenerative destruction of the IVD, the endplates, and the vertebral bodies [7]. DDD is a complex degenerative process due to age-related changes in molecular composition of the disc. This cascade has biomechanical and often times clinical sequelae that can result in substantial impairment in the afflicted individual.

### 2.3. Genetic Component of Degeneration

An undeniable genetic component to degenerative disc disease becomes evident when looking at results from twin studies and studies involving mice with a knockout for genes suspected to play a role in disc degeneration [8, 9]. Among the genes suggested to be involved in DDD, are genes that code for collagens I, IX, and XI, interleukin 1 (IL-1), aggrecan, the vitamin D receptor, matrix metalloproteinase 3 (MMP-3), and other proteins [10]. It is well recognized that DDD is regulated by these and many other genes. Interactions among those genes, which in concert contribute substantially to DDD despite presumably small individual contributions, as well as gene-environment interactions, are very likely [11].

### 2.4. Environmental Factors

Many practitioners believe environmental factors to be a secondary consideration to the genetic component of DDD. Nevertheless, the influence of environmental factors on DDD is far from negligible and has been defined in a comprehensive manner by Williams and Sambrook in 2011 [12]. In a meta-analysis, odds ratios for manual materials handling, frequent bending or twisting, and whole-body vibration were calculated to be 1.51, 1.68, and 1.39 in regard to DDD, respectively [13]. A modest association between smoking and disc degeneration has been shown, suggesting possible influences of chemical exposures [14]. Twin [15] as well as animal studies [16] have postulated an involvement of nicotine in disc degeneration, which might be due to impaired blood flow to the disc [17]. Furthermore, an association of atherosclerotic lesions in the aorta and LBP, reflecting a possible link between atherosclerosis and DDD, has been reported [18].

## 3. Clinical Presentation

Patients with lumbar disc disease often present with a myriad of symptoms including pain, radicular symptoms, and weakness. LBP may be exacerbated by position and movement. Flexion often worsens the symptoms, while extension will relieve them. An increase in pain with extension may indicate facet arthropathy.

When examining patients with presumed lumbar DDD, it is important to exclude other potential known etiologies for their pain. Abdominal pathology including aortic aneurysms, pancreatic disease, and renal calculi must be excluded. Furthermore, it is imperative that patients be questioned regarding other symptoms such as fevers, chills, fatigue, and weight loss, which may be indicative of other pathology.

## 4. Diagnosis

Upright plain radiographs in two planes are the initial imaging study of choice. They aid in ruling out pathologies such as deformity, fractures, or metastatic cancer as underlying causes of back pain and, often supplemented by other imaging modalities, are evaluated for signs of degeneration. Findings in degenerative discs include disc space narrowing, endplate sclerosis, “vacuum” phenomenon within the disc, and osteophytes. Flexion and extension views may be helpful if instability is suspected.

**Figure 1:**
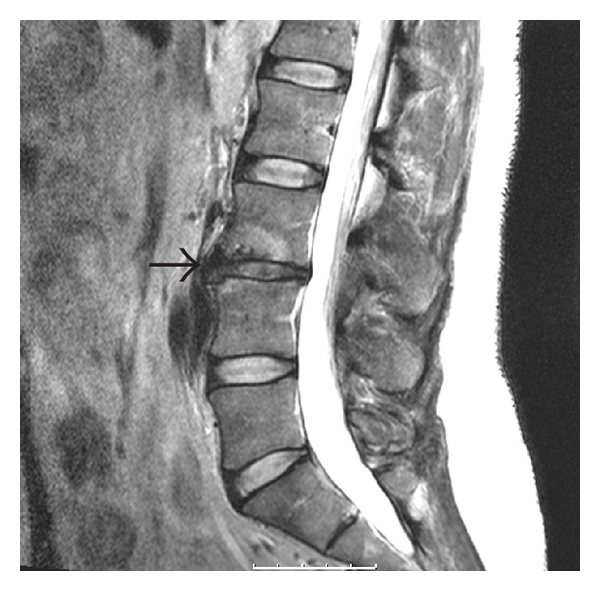
Disc space narrowing and degenerative changes at the L3-L4 level (arrow) on sagittal T2-weighted MRI.

Magnetic Resonance Imaging (MRI) is a more sensitive imaging study for the evaluation of degenerative disc disease. Findings on MRI scan include disc space narrowing, loss of T2 signal within the nucleus pulposus, endplate changes, and signs of internal disc derangement or tears (Figure 1). High Intensity Zones (HIZ) have been found in close to one third of patients undergoing MRIs for low back pain and have been used as a marker for internal disc derangement. However, the accuracy and reliability of these HIZs has been questioned [19, 20].

Modic et al. were among the first to radiologically characterize vertebral endplate changes that are associated with degenerative disc disease [21, 22]. The Modic classification system includes three types of changes, and grading has been shown to be reliable and reproducible [23]. In Type I, there is increased signal on the T2-weighted sequence and decreased signal intensity on the T1 sequences indicative of marrow edema. Type II is characterized by fatty infiltration of the marrow as demonstrated by hyperintense T1 and T2 images. Finally, Type III demonstrates hypointense signals on T1 and T2 sequences, which corresponds to endplate sclerosis. The Modic types are summarized by Table 1.

 Pfirrmann et al. further examined and characterized intervertebral disc pathology using MRI [24]. The degree of disc degeneration were graded I through V. Grade I discs are white, and homogenous on T2 sequences. Grade II discs are white, but somewhat inhomogenous with banding. Grade III discs are grey with unclear distinction between the nucleus and annulus. Grade IV discs are inhomogenous and dark without distinction between the nucleus and annulus. Finally, Grade V discs demonstrate a collapsed disc space. The Pfirrmann grading system is depicted by Table 2.

 While plain radiographs and MRI provide information regarding the health of the intervertebral segment, they do not provide any information regarding the segments impact on clinical symptoms. The use of discography has attempted to identify specific degenerated discs as pain generators [25]. Provocative discography involves the injection of contrast dye into the nucleus. Computed tomography is used to evaluate for extravasation of dye indicating annular tears. The patient's symptoms and intradiscal pressure during the injection are also recorded. If the pain on injection is similar to their back pain, then the discogram is considered concordant. Also, if pain is produced at low pressures, it is felt that there is symptomatic annular disruption or internal derangement. However, if the pain is different or produced at high pressures of injection, the test is often considered discordant. Still, low-pressure discography has been found to have false positive rates of up to 25% in asymptomatic individuals and may accelerate disc degeneration [26, 27].

## 5. Treatment Strategies for Lumbar Degenerative Disc Disease

### 5.1. Mechanical Concepts of Lumbar Disc Regeneration

Spinal fusion surgery is a recognized treatment option of LBP but its efficacy and success remain controversial. It can be achieved by a variety of approaches and techniques, including posterolateral fusion, anterior lumbar interbody fusion, and posterior lumbar interbody fusion. Minimally invasive approaches to the lumbar spine for interbody fusion, such as lateral lumbar interbody fusion, have been gaining popularity within the last 5 years [28].

While fusion procedures offer a way of eliminating motion between spinal segments, and thus alleviate discogenic pain associated to degenerative changes, they address only a symptom and not the cause of DDD. Furthermore, there are significant concerns regarding alterations in adjacent segment motion, which may lead to the introduction of adjacent segment degeneration [29–31]. As a result, motion preserving procedures have been introduced to assist in preventing adjacent segment changes. Disc arthroplasty has the purported advantage of removing the degenerated intervertebral disc and replacing it with a prosthesis that will allow motion between the segments. Clinical trials have shown equivalent results compared with circumferential fusion for the treatment of discogenic pain [32]. In a two-year follow-up study, total disc replacement patients compared favorably to an arthrodesis control group in terms of pain relief and recovery, but a potential early time point patient bias in favor of the arthroplasty group necessitates a longer followup and concern was expressed in regard to long-term polyethylene wear in total disc replacements with a polyethylene component [33]. Furthermore, the purported advantages of preventing adjacent segment disease are unclear and require additional long-term results [34].

Another potential motion-preserving surgery involves posterior dynamic stabilization. These systems involve placement of pedicle screws across a motion segment connected by a flexible graft. These devices are designed to restrict motion across the interspace to limit discogenic pain [35]. Early followup of this technique has demonstrated some promising result in the treatment of discogenic back pain with regard to improved VAS and ODI scores [36, 37]. However, longer-term studies have demonstrated adjacent segment disease in 29–47% of patients [38–40].

### 5.2. Cell-Based Therapies and Growth Factors in Lumbar Disc Degeneration

While there are a variety of invasive, surgical options for the treatment of lumbar degenerative disc disease, recent emphasis has been directed at the reversal of disc degeneration or the replacement of the affected disc. Various therapies have been investigated including biologic growth factors, stem cells, and gene transplant. While these novel therapeutic modalities have shown some early promising results with regards to reversal of the degenerative cascade, their clinical effects and long-term results are uncertain [41]. It is also unclear, whether differentiation of stem cells into mature tissues may cause them to express immunogenic markers, which ultimately may result in stem cell rejections.

 In 2002, Bone Morphogenetic Protein (BMP) was approved as a bone graft substitute for anterior lumbar interbody fusion (ALIF), but in addition to its osteoinductive properties, BMP also demonstrated some potential for the treatment of disc disease [42]. Current human and animal studies have shown upregulation of BMP-2 and -7 in aging discs. This upregulation has been found to have an antiapoptotic effect on the cells of the nucleus pulposus [43]. Also, the introduction of BMP-2 into intervertebral discs has resulted in increased extracellular matrix production [44]. However, the direct introduction of BMP into the intervertebral disc may lead to potential undesired osteogenic effects. In recent years, concerns about the safety of BMP-2 have arisen following reports of adverse reactions attributable to its use in ALIF and its off-label use in other spinal fusions [45–47]. In 2008, the FDA published a public health notification about potentially life-threatening complications associated with use of BMP in cervical spine fusion [48]. To date, the safety of recombinant BMP-2 as a bone graft substitute remains controversial. Recent studies have shown the potential for the drug simvastatin to induce chondrogenesis and the production of Type II collagen and aggrecan through BMP-mediated pathways [49].

Transplantation of stem cells has emerged as another promising treatment strategy for DDD [40, 50–52]. Recent animal studies have shown increased extracellular matrix when autologous disc-derived chondrocytes were introduced into a canine disc degeneration model. Furthermore, recent human trial involving the introduction of autologous chondrocytes into postdiscectomy patients has resulted in decreased pain at 2 years compared with controls. Also, there was increased disc hydration at the treated levels and adjacent levels as evidenced by MRI evaluation [53].

 An alternative technique to chrondrocyte transplantation has been the use of adipocyte progenitor cells. The advantage to this technique is the relative abundance of adipose-derived stem cell when compared to chondrocytic stem cells. In a rat degenerative disc disease model, transplanted adipose-derived stem cells resulted in increased extracellular matrix production, minimally decreased disc height, and improved discal hydration when compared to controls [54].

 Finally, another promising type of stem cells for future investigation are bone-marrow-derived stem cells. *In vitro *studies have demonstrated that these cells have similar chondrogenic capacity when compared to nucleu-pulposus-derived cells [55]. However, *in vivo* studies are needed to confirm their potential efficacy, and any strategy involving the introduction of new cells into the human intervertebral disc to induce regeneration would have to account for the increased demand of nutritional supply by the increasing number of cells or the increased activity of previously present cells [56].

### 5.3. Gene Therapy in Lumbar Disc Degeneration

Transduction of genes that have the potential to interfere with disc degeneration or even induce disc regeneration is a concept recently applied to DDD by researchers. This strategy requires identification of relevant genes that play a role in the disc degeneration cascade, as well as ways of delivering those potentially therapeutic genes into disc cells. This can be obtained by so-called gene vector systems, which include a variety of viral and, more recently, nonviral vectors [57]. Safety issues are imminent to the use of vectors, and absence of adverse effects is imperative to any vector system.

Early studies used viral vectors to deliver marker genes into discs *in vitro* and *in vivo* [51, 58]. The first gene with potentially beneficial effects on disc degeneration to be experimentally delivered to the IVD in an animal model was TGF-*β*1 [59]. A similar approach of initial transduction of a marker gene was taken by Moon et al. to deliver genes into human IVD cells [60].

 Additionally, other growth factors [61], inhibitors of metalloproteinases [62], and also a transcription factor, Sox-9 [63], have received consideration as possible targets for gene therapy for DDD. Following identification of ADAMTS5 as a contributor to cartilage degradation in a mouse model [64], ADAMTS5 small interference RNA was successfully used in a rabbit model to suppress degradation of NP tissue [65]. A similar approach was used to target caspase 3, a main executor of apoptosis, in a rabbit model [66]. Future *in vivo* studies linking theoretical benefits of any of these gene therapy approaches to situations possibly encountered in clinical practice are desirable [67] and comprise the long-term perspective of applying gene therapy as a strategy to treat the underlying mechanism of disc degeneration.

### 5.4. Summary

Degenerative lumbar disc disease and resulting low back pain impart a large socioeconomic impact on the health care system. Disc degeneration is a multifactorial occurrence with a strong genetic component. Age and environmental factors contribute to the degenerative process. While current strategies aim to remove the pain generator through surgery, future, emerging modalities aim to reverse the degenerative cascade through the use of biologics and gene modification. Advances in fields such as genomics, nanotechnology, stem cell biology, gene therapy, and tissue engineering have tremendous therapeutic potential for clinical applications in degenerative disorders such as DDD, but novel treatment strategies for lumbar disc degeneration require further evaluation in preclinical and clinical trials.

## Figures and Tables

**Table 1:** Modic changes as illustrated by Jones et al. [23].

Type	T1 MRI signal intensity	T2 MRI signal intensity
I	hypointense	hyperintense
II	hyperintense	iso- or hyperintense
III	hypointense	hypointense

**Table 2:** Pfirrmann grades as illustrated by Pfirrmann et al. [24].

Grade	Structure	Distinction (nucleus and annulus)	T2 MRI signal intensity	Disc space height
I	white, homogenous	clear	isointense to cerebrospinal fluid (hyperintense)	normal
II	inhomogeneous, with banding	clear	isointense to cerebrospinal fluid (hyperintense)	normal
III	gray, inhomogeneous	unclear	intermediate	normal to decreased
IV	gray to black, inhomogenous	no distinction	intermediate to hypointense	normal to decreased
V	black, inhomogenous	no distinction	hypointense	collapsed
